# Expanding the clinical phenotype of HIST1H1E syndrome: cerebellar atrophy and bilateral optic neuropathy, a case report

**DOI:** 10.3389/fneur.2026.1820525

**Published:** 2026-06-03

**Authors:** Zinnia Sen Yen Lai, Nicola O'Neil, Ezann Siebert, Stephen Colley, Peter K. Panegyres

**Affiliations:** 1 Sir Charles Gairdner Hospital, Nedlands, WA, Australia; 2 Neurodegenerative Disorders Research Pty Ltd, West Perth, WA, Australia; 3 Genetic Health WA, Subiaco, WA, Australia; 4 Ophthalmology Department, Fremantle Hospital, Fremantle, WA, Australia; 5 Western Eye, Mosman Park, WA, Australia; 6 School of Medicine, The University of Western Australia, Nedlands, WA, Australia

**Keywords:** cerebellar degeneration, HIST1H1E syndrome, histone variants, optic nerve atrophy, Rahman syndrome

## Abstract

**Background:**

HIST1H1E syndrome is caused by frameshift variants in the *HIST1H1E* gene; while strabismus and refractive errors have been previously reported, this is the first case describing optic and cerebellar atrophy in an affected individual to our knowledge.

**Methods:**

A 51-year-old woman with intellectual disability, recurrent falls, and progressive visual difficulties required comprehensive ophthalmic, neurologic, and genetic evaluation.

**Results:**

Systemic examination revealed characteristic facial dysmorphism, brachydactyly, ataxic gait, tremor, and positive Romberg's sign. Ophthalmic examination showed bilateral optic disc pallor with patchy visual field deficits. Optical coherence tomography (OCT) confirmed retinal nerve fiber involvement and ganglion cell layer (GCL) loss. Electrophysiology supported bilateral optic neuropathies. Computed Tomography demonstrated optic nerve and cerebellar atrophy. Whole-exome sequencing identified a pathogenic heterozygous variant c.464dupC.p.(Lys157Glufs^*^39) in the *HIST1H1E* gene.

**Conclusion:**

This patient expands the HIST1H1E syndrome phenotype to include cerebellar and optic nerve atrophy compatible with a neurodegenerative process.

## Introduction

HIST1H1E syndrome (previously known as Rahman syndrome; OMIM #617537) is a rare neurodevelopmental disorder caused by *de novo* truncating variants in the *HIST1H1E* (also known as *H1-4)* gene on chromosome 6p22.2 which was first described in 2017 (1, 2). This gene encodes histone H1.4, one of the linker histones involved in chromatin compaction and gene regulation (1, 11–14). Around 50 cases have been previously reported, with variants clustering in a small 94-base pair region of the C-terminal domain (1, 13). At present, all variants identified in affected individuals have been *de novo* and heterozygous frameshift variants within the C-terminal domain of HIST1H1E (15) consistent with an autosomal dominant disorder. Affected individuals have a variable phenotype with significant heterogeneity. Most atypically present with intellectual disability and somatic overgrowth. Other commonly reported features can include hypotonia, distinctive facial gestalt (full cheeks and high hairline in childhood evolving to frontal bossing and deep-set eyes in adulthood, aged appearance, prominent cheek bones, wide nasal bridge), behavioral issues and skeletal abnormalities, endocrine abnormalities particularly hypothyroidism, craniofacial anomalies and ectodermal changes (1–14).

Pathogenic *HIST1H1E* variants disrupt the C-terminal tail of histone H1.4, which undergoes cell cycle-dependent phosphorylation to regulate chromatin structure (2, 12, 13). Without these regulatory sites, cells show abnormal chromatin compaction and widespread transcriptional disruptions (1, 13). Flex et al. demonstrated that *HIST1H1E* mutant cells have accelerated cellular senescence, chromosomal instability, defective DNA repair, telomere dysfunction, and features resembling premature aging (15). Taken together, this suggests that truncating *HIST1H1E* variants lead to the clinical features associated with HIST1H1E syndrome of neurodevelopmental problems, osteoporosis and progressive dental disease (1, 15). Based on the current reported cases, there appears to be no obvious genotype-phenotype correlation with patients and significant variability and heterogeneity in presentation and severity (10).

Previously, reports of ocular involvement in individuals with HIST1H1E syndrome has been limited to strabismus, amblyopia, ocular deformities and refractive errors (1, 10, 12, 13). We describe, for the first time to our knowledge^*^, a patient with bilateral optic nerve atrophy, retinal ganglion cell loss, and marked cerebellar atrophy with whole exome sequencing (WES) and features consistent with a diagnosis of HIST1H1E syndrome.

## Case presentation

A 51-year-old female with intellectual disability presented with a 12-month history of recurrent falls and difficulty with her lower visual fields. Medical history included well-controlled epilepsy, bilateral cataracts, anxiety, hypothyroidism, osteoporosis and kyphosis, muscle weakness, tooth decay and enamel hypoplasia. Bilateral cataract surgery was performed 20 years prior with no complications. She had minimal refractive error (right eye: sphere −0.25 and cylinder −0.50; left eye: sphere +0.50 and cylinder −0.75). There was no history of significant head or orbital trauma. The patient had no history of alcohol consumption, and there was no family history of neurological conditions. She lived in a group home with her sister as her primary carer.

On examination, height was 149 cm (below 3rd percentile), weight 58.2 kg and head circumference 55.5cm (50th percentile). The patient had distinctive facial features including full cheeks, long philtrum, thin upper lip, downturned mouth corners, arched eyebrows, high frontal hairline, and bitemporal narrowing (Figure 1a). She had brachydactyly, nail dysplasia, and widely spaced teeth (Figure 1b).

**Figure 1:**
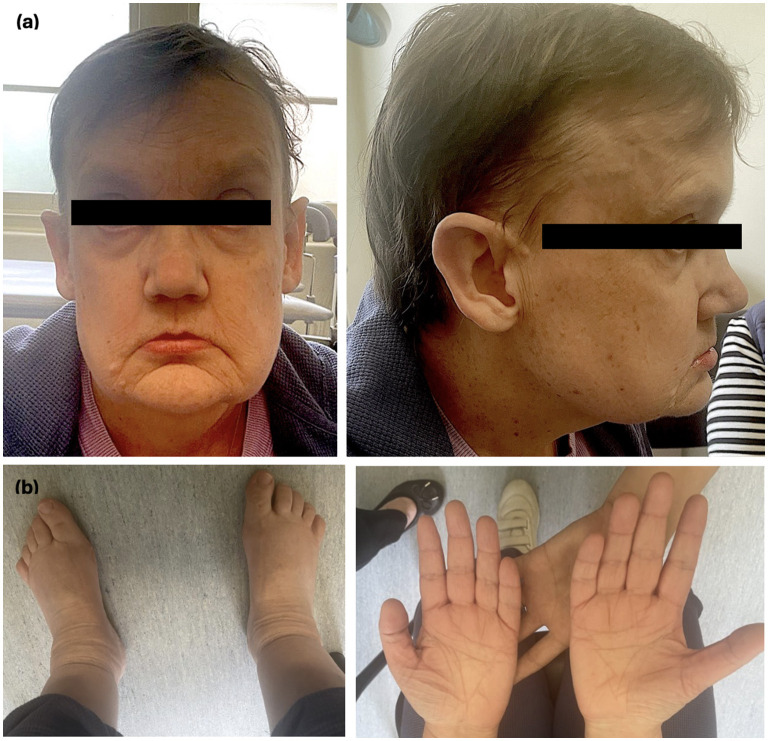
Facial and limb features. **(a)** Facial features include high frontal hairline, full cheeks, bitemporal narrowing, sparse arched brows, and low-set ears. **(b)** Limb images show brachydactyly of the toes and fingers.

Neurological assessment revealed a wide-based ataxic gait, mild postural and kinetic tremor, and positive enhanced Romberg's sign.

Computed tomography (CT) scan of the brain showed cerebellar atrophy, maximal in the vermis and bilateral optic nerve atrophy (Figure 2). No compressive lesions, retro-orbital masses or other structural abnormalities were identified. Magnetic resonance imaging (MRI) was declined by the patient and her guardian. EEG revealed right temporal and posterior epileptogenic activity. Audiometry confirmed moderate-severe sensorineural hearing loss. No other causes of optic neuropathy, retinal ganglion cell pathology and cerebellar atrophy were identified, including toxic, metabolic, paraneoplastic, autoimmune, vascular, infectious, trauma or nutritional.

**Figure 2:**
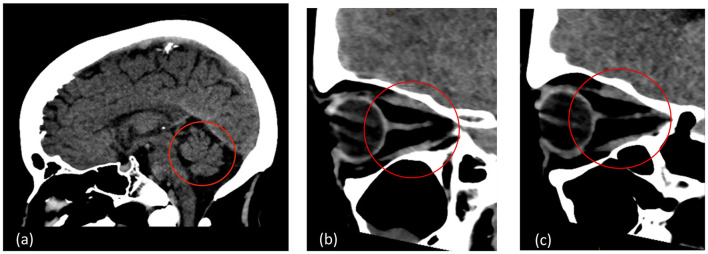
Sagittal view of a CT scan of the brain **(a)** cerebellar atrophy, **(b)** right optic nerve atrophy, **(c)** left optic nerve atrophy. Note the reduced cerebellar size in **(a)**, and the thinning of the optic nerves in **(b)** and **(c)**.

### Ophthalmic assessment

Best corrected visual acuity (VA) of 6/9 (right) and 6/12 (left) (Snellen chart), similar to measurements 20 years earlier, 6/9 bilaterally. Confrontation visual fields showed asymmetric inferior field loss, with the left eye more affected than the right eye. Ishihara color vision testing was reduced in the right eye (8/17 plates). There was no ocular manifestation of cerebellar dysfunction. Ocular motility examination revealed full extraocular movements in all directions of gaze. Cover-uncover testing demonstrated orthophoria in primary gaze. No nystagmus was observed. Slit-lamp examination revealed thin corneas, with central corneal thickness measuring 435 μm (right) and 454μm (left). Intraocular pressures (IOP) were 16 mmHg and 11 mmHg, respectively. Fundoscopy showed bilateral optic nerve pallor. Optical Coherence Tomography (OCT) confirmed significant bilateral retinal nerve fiber layer and ganglion cell layer (GCL) thinning (Figures 3a, b) consistent with optic atrophy, with macular ganglion cell layer (GCL) on OCT demonstrating diffuse bilateral thinning and marked central involvement (14 μm right, 11 μm left; Figures 3c, d). The maculae were otherwise structurally preserved with no outer retinal abnormalities. Esterman binocular visual field testing revealed moderate peripheral visual field loss with a horizontal extent of 80°, and patchy bilateral inferior visual field loss (Figure 4a). Standard automated perimetry provided very inaccurate results despite multiple attempts. Given the thin corneas and possible underestimation of IOP, along with her optic disc appearance, empiric topical antiglaucoma treatment was started (Latanoprost 0.005%) to both eyes at night; subsequent IOP was 10 mmHg (right) and 11 mmHg (left).

**Figure 3:**
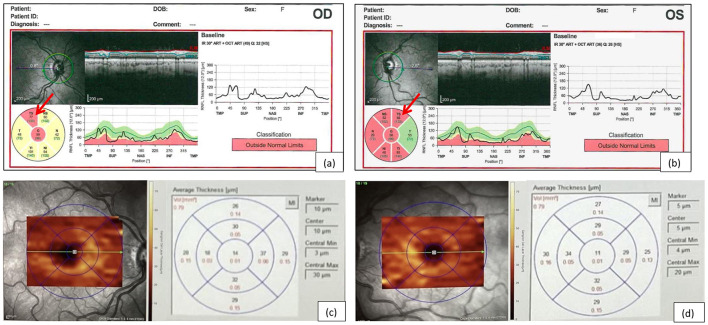
Infrared (IR) 30° and automated real-time (ART) OCT images of both eyes, Right eye **(a)** and Left eye **(b)**, showing marked optic nerve atrophy with superior retinal nerve fibre layer (RNFL) and ganglion cell layer (GCL) loss, stable over time. Note red arrows showing areas with significant RNFL thinning. Macular GCL OCT demonstrating diffuse bilateral ganglion cell layer thinning, with marked central involvement (central thickness: 14 um right, 11 um left) and surrounding macular values 18–37 um, consistent with severe central ganglion cell loss in both eyes. Right eye **(c)**, and left **(d)**.

**Figure 4:**
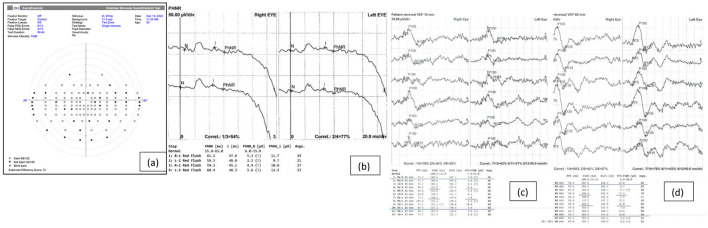
Functional studies: Esterman field **(a)** showing horizontal extent greater than 80° with temporal deficits, patchy lower field losses. Visual electrophysiology demonstrating bilaterally reduced photonegative response (PhNR) **(b)**. Pattern-reversal visual evoked potentials (VEP) using large **(c)** and small **(d)** check sizes, demonstrating essentially normal responses bilaterally.

Electroretinography (ERG) demonstrated bilaterally reduced photopic negative response (Figure 4b). Visual Evoked Potentials (VEP) were essentially normal (Figures 4c, d).

### Genetic investigation

Chromosomal microarray analysis did not identify any significant copy number variants. Whole exome sequencing (WES) was performed on a buccal sample through Blueprint Genetics. The analysis included sequencing of all protein-coding genes, whole exome copy number variant analysis, and mitochondrial genome sequencing.

WES identified a heterozygous frameshift variant in HIST1H1E c.464dupC, p.(Lys157Glufs^*^39) (ClinVar Accession: VCV000635261.6). This variant is absent in the Genome Aggregation Database (gnomAD) and generates a frameshift resulting in a premature stop codon in the only protein coding exon. This variant has been reported previously in at least one other individual with HIST1H1E syndrome, who was diagnosed in childhood after presenting with overgrowth, focal seizures, autism spectrum disorder and facial dysmorphism (15). They had no significant eye abnormalities and last underwent a MRI brain at the age of 2 years old which was unremarkable. As per the American College of Medical Genetics and Genomics guidelines (16), this variant is classified as pathogenic.

No other relevant variants of uncertain significance were identified on WES.

## Discussion

To our knowledge, this is the first reported patient with HIST1H1E syndrome with bilateral optic nerve atrophy, retinal ganglion cell pathology, and cerebellar atrophy^*^. The identified variant (c.464dup, p.Lys157Glufs^*^39) is known disrupt the C-terminal region of histone H1.4, which is critical for chromatin binding and cell cycle regulation (1, 15), but the neurological phenomena described herein are new and not previously reported^*^.

The optic nerve damage likely reflects accelerated neurodegeneration, as *HIST1H1E* variants cause genomic instability, defective DNA repair, and premature cellular senescence (15). Retinal ganglion cells may be particularly vulnerable due to their high metabolic demand (17). While previous reports documented strabismus and refractive errors (1, 12), structural optic nerve pathology represents a novel finding.

The differential diagnosis warrants consideration of glaucoma, given the optic nerve cupping pattern and thin corneas (which could lead to underestimation of IOP). However, several features argue against primary open-angle glaucoma as the sole explanation: First, the profound loss of ganglion cells on OCT with normal IOP (RE:16mmHg and LE:11mmHg), even allowing for thin corneas, would not be a typical finding in glaucoma. Second, the reduced photopic negative response on ERG indicates ganglion cell dysfunction, while VEPs were essentially normal. The preservation of VEP conduction despite significant structural loss on OCT indicates that the residual ganglion cells maintain relatively normal axonal function while the overall cell population is depleted, a pattern characteristic of chronic neurodegenerative processes affecting ganglion cell bodies (17). This suggests that the pathology is primarily at the retinal ganglion cell level rather than the posterior visual pathway.

Third, the combination of bilateral symmetric ganglion cell pathology, cerebellar atrophy and systemic premature aging features in the setting of confirmed HIST1H1E pathogenic variant suggests a neurodegenerative process. Nevertheless, given thin corneas and optic nerve damage, a contributory glaucomatous component cannot be definitively excluded, which is why prophylactic anti-glaucomatous therapy was initiated.

The cerebellar atrophy correlates with progressive ataxia, tremor, and positive Romberg's sign. Cerebellar neurons may be similarly vulnerable to accelerated senescence and genomic instability (15). Progressive neurodegeneration has been described in other chromatin remodeling disorders and premature aging syndromes (18, 19). Consistent with this, our patient exhibited multiple premature aging features previously identified by Flex et al., including severe osteoporosis, progressive kyphosis, sensorineural deafness, extensive dental disease, and progressive neurologic decline (15). The accelerated cellular aging, nuclear lamina abnormalities, elevated p53, telomere dysfunction, and premature senescence observed in HIST1H1E-mutant cells provide a pathophysiological basis for this progressive neurodegeneration and mirror mechanisms described in classical progeroid disorders such as Hutchinson-Gilford progeria and Werner syndrome (18, 19). The proposed mechanism of histone dysfunction resulting in neurodegeneration in this patient expands our comprehension of neurodegenerative processes.

A thorough work-up was undertaken to exclude alternative causes of combined retinal ganglion cell pathology and cerebellar atrophy. Full blood count, vitamin B12, folate, angiotensin-converting enzyme, anti-CCP antibody, and CRP levels were within normal limits. Syphilis and tuberculosis serology, ANCA, PR3 and MPO were negative. ANA demonstrated a low-titer nucleolar pattern (1:160) with a cytoplasmic staining component, and rheumatoid factor was elevated (116; normal <30); however, in the absence of systemic symptoms, inflammatory markers, myositis features, or other supportive autoimmune serology, these findings were considered non-specific and not indicative of an underlying autoimmune process. The patient reported no alcohol consumption, excluding toxic optic neuropathy and alcohol-related cerebellar degeneration. Overall, no evidence of mitochondrial disease, metabolic disorders, autoimmune or infectious etiologies, vascular pathology, or nutritional deficiencies was identified that could account for the observed neuro-ophthalmic and cerebellar findings.

The major strength of this report is that our patient is one of the oldest individuals reported with HIST1H1E syndrome, enabling characterization of late-onset and progressive manifestations that may not be evident in childhood cohorts. Previous reports have largely involved pediatric patients, with only limited adult cases described (1). The detailed multimodal ophthalmic and neuroimaging characterization performed on our patient allows comprehensive structural correlation with neurological decline and expands the phenotypic spectrum to include optic neuropathy with retinal ganglion cell loss and cerebellar degeneration. However, several imitations should be acknowledged. Firstly, this is a single-case observation, and causality between the *HIST1H1E* variant and the observed neurodegeneration cannot be definitively established. Secondly, neuroimaging was limited to CT scanning as the patient and her guardian declined MRI. While CT identified cerebellar atrophy and excluded compressive lesions or other structural abnormalities, MRI would have provided superior characterization of the optic nerves, optic chiasm and cerebellar structures. Thirdly, the optic nerve cupping with associated RNFL and GCL loss raises the possibility of contributory glaucomatous optic neuropathy. This cannot be definitively excluded, although the combination of symptoms and signs suggests a neurodegenerative process.

## Data Availability

The datasets presented in this article are not readily available because of ethical and privacy restrictions. Requests to access the datasets should be directed to the corresponding author.
