# Supplementary material for: Expanding the clinical phenotype of HIST1H1E syndrome: cerebellar atrophy and bilateral optic neuropathy, a case report
Source: Front Neurol. 2026 Jun 3;17:1820525. doi: 10.3389/fneur.2026.1820525 (PMC13272026; doi:10.3389/fneur.2026.1820525)
Supplement: Supplementary file 1 [file Data_Sheet_1.docx]

**Supplementary material:**

**HIST1H1E Syndrome – Optic Atrophy and Cerebellar Atrophy**

**Patient impact statement**

My sister has had an intellectual disability and epilepsy from birth. We as a family have always adapted and adjusted to support her on her life journey.

In 2023 my sisters balance became a concern as she sustained five falls that year, she was referred to Neurologist: Professor Panegyres for investigation. My sister was diagnosed with Optic Atrophy and Cerebellar Atrophy which has affected both her vision and her balance and after genetic testing she was also diagnosed with HIST1H1E syndrome.

These diagnoses have significantly impacted my sister and our family as we adjust to her new circumstances. Over the past year we have seen a significant change in her support needs, this decline has been incredibly difficult to accept. My sister knows that she is not the same girl she used to be and really struggles to process and understand what is happening to her. Her balance has declined to the point that she now requires the use of a four-wheel walker and a shower chair to ensure her safety.

The Optic Atrophy has significantly affected her daily living, and the decline of her peripheral vision has been frightening for her and emotionally distressing for our family watching these very significant changes and how they affect her daily living. My sister has always navigated life visually, meaning that she takes her clues and cues from others by reading facial expressions and body language as part of the strategy she uses to communicate with others. She has said that she is ‘scared’ about losing her sight. She did have corrective cataract surgery to both eyes in April and May 2003 when she was 31 years old. After the surgery I asked the Ophthalmologist why she had developed cataracts at such a young age his reply was “I’m not sure but I would suspect that whatever caused the disability caused the cataracts”.

The issues arising from the HIST1H1E Syndrome, the Optic Atrophy and the Cerebellar Atrophy are many. My sister’s world has always been small because of her Intellectual Disability but over the last year in particular her world has further diminished. Her confidence in herself and her abilities is declining as Optic Atrophy and Cerebellar Atrophy progress.

Our reason for participating in this research paper is to have a better understanding of her diagnosis and an insight into how we can help her to manage the effects as her symptoms progress. We also want this information to be made available for individuals and family members who are living with a HIST1H1E diagnosis.
